# Some like it hot: *Candida* activation of inflammasomes

**DOI:** 10.1371/journal.ppat.1008975

**Published:** 2020-10-29

**Authors:** Giorgio Camilli, James S. Griffiths, Jemima Ho, Jonathan P. Richardson, Julian R. Naglik

**Affiliations:** Centre for Host-Microbiome Interactions, Faculty of Dentistry, Oral and Craniofacial Sciences, King’s College London, London, United Kingdom; University of Maryland, Baltimore, UNITED STATES

## Introduction

*Candida albicans* is a polymorphic fungus that causes a wide spectrum of complex diseases ranging from superficial mucocutaneous disorders to life-threatening invasive and disseminated infections, particularly in immunocompromised individuals. Understanding this complex *Candida*–host interaction and the mechanisms that favour protective immunity over immune pathology may provide valuable insights for the rational design of new immunotherapies. Immunity to *C*. *albicans* during mucosal and disseminated infections involves the cooperative action of innate and adaptive immune effectors that ultimately determines patient outcome. In recent years, huge progress has been made in our understanding of *Candida* immunity, particularly with regard to inflammasome activation, characterised by the release of the cytokines interleukin (IL)-1β and IL-18.

IL-1β and IL-18 are potent pro-inflammatory cytokines that coordinate the activation of innate and adaptive immune cells. They are produced as inactive cytoplasmic precursors (pro-IL-1β and pro-IL-18) in response to danger- or pathogen-associated molecular patterns (DAMPs/PAMPs; priming step) and must be posttranslationally processed by multimeric complexes, termed ‘inflammasomes’, to generate the mature, biologically active cytokines [1]. Inflammasomes typically consist of a sensor protein, an adaptor (apoptosis-associated speck-like protein containing a caspase recruitment domain (CARD) (ASC)), and an effector caspase. Inflammasome assembly and activation of downstream caspases are triggered by several molecular and cellular signalling events (activation step) that include ion flux, reactive oxygen species (ROS), mitochondria dysfunction, or lysosomal destabilisation [1]. In addition to cytokine secretion, pro-inflammatory caspases localised on inflammasomes can also cleave and activate the pore-forming protein gasdermin D (GSDMD). Cleaved GSDMD oligomerises and forms pores in the plasma membrane, leading to an inflammatory form of programmed cell death called pyroptosis, which functions as a host defence mechanism in a wide range of microbial infections [2]. However, inflammasome activation may be a double-edged sword and thus requires tight regulation to be protective rather than immunopathogenic [3]. Accordingly, determining the role played by inflammasomes during infectious diseases will be essential to identify intervention strategies aimed at boosting or inhibiting inflammasome-mediated immune responses.

## Inflammasome activation during *C*. *albicans* infection

Multiple inflammasomes can be activated as a result of the complex interplay between host receptors and *C*. *albicans* cell wall components and secreted molecules. Thus far, *C*. *albicans* has been shown to activate nucleotide-binding oligomerisation domain (NOD)-like receptor family pyrin domain-containing 3 (NLRP3), NOD-like receptor family CARD domain-containing protein 4 (NLRC4), and noncanonical/caspase-8 or caspase-11 inflammasomes in myeloid or epithelial cells (see *PLOS Pathogens* Pearls [4] for further details and references). Furthermore, accumulating evidence has demonstrated that *Candida*-induced inflammasome activation can lead to pyroptotic cell death (see *PLOS Pathogens* Pearls [5] for further details and references). Although the precise signalling sensed by cytosolic NLRs remains unclear, several molecular and cellular events leading to *Candida*-induced inflammasome activation are emerging. Fungal cell wall components can be recognised by cell surface C-type lectin receptors (CLRs) and Toll-like receptors (TLRs), which mainly provide a priming signal leading to the synthesis of pro-IL-1β and NLRP3 inflammasome components. For instance, engagement of the CLR dectin-1 and TLR2 by β-glucan and zymosan has been demonstrated to prime macrophages for subsequent IL-1β release by *C*. *albicans*, whilst the 2 stimuli were unable to elicit this response by themselves [6]. Similarly, Myd88 and B cell lymphoma 10 (BCL10), which mediate signals downstream of TLRs and CLRs, respectively, have been recently demonstrated to be necessary (induction of NLRP3 and IL-1β transcripts) but not sufficient to trigger inflammasome activation and pyroptosis in macrophages, suggesting that inflammasome activation and priming can be decoupled in response to *C*. *albicans* [7]. However, *C*. *albicans* activation of spleen tyrosine kinase (Syk)-coupled CLRs was found to induce both pro-IL-1β synthesis and NLRP3 inflammasome activation through ROS production and potassium efflux in murine dendritic cells [8]. Furthermore, β-glucan and both heat-killed and live *Candida* can induce the assembly and activation of a noncanonical CARD-9/BCL10/mucosa-associated lymphoid tissue lymphoma translocation (MALT)-1/ASC/caspase-8 inflammasome in human dendritic cells independently from dectin-1 internalisation or an intracellular NLR [9]. This alternative pathway may represent a rapid mechanism through which human dendritic cells release IL-1β to initiate and polarise appropriate adaptive immune responses. These findings also suggest that different signalling pathways may lead to inflammasome activation in distinct mononuclear phagocyte subsets during *C*. *albicans* infection.

After recognition of *Candida* PAMPs, host cell surface receptors mediate the internalisation of the pathogenic fungus. Phagocytosis of *C*. *albicans* and host lysosomal dysfunction were shown to be required for NLRP3 inflammasome activation [10]. Indeed, treatment of macrophages with cytochalasin D or CA-074-Me, which inhibit phagocytosis and cathepsin B, respectively, inhibited IL-1β production following *C*. *albicans* infection [10]. Furthermore, the yeast-to-hypha transition [10] and changes in the profile of PAMPs expressed in the hyphal cell wall, for example, the absence of a fully matured, branched outer mannan layer and the exposure of inner hyphal cell wall components (e.g., β-glucan and chitin), have been proposed to enhance recognition by pattern recognition receptors (PRRs) and IL-1β production by macrophages [11]. Recently, however, the importance of *C*. *albicans* morphogenesis per se in the induction of inflammasome activation and pyroptosis has been placed under scrutiny. Indeed, filamentation-deficient *C*. *albicans* mutants capable of triggering IL-1β secretion and pyroptosis [12,13] and *C*. *albicans* mutants that retain the ability to form hyphae but induce decreased IL-1β secretion and macrophage pyroptosis [13,14] have been identified. These findings have also highlighted that biochemical features of the fungal cell wall, rather than just the physical morphology of *Candida*, likely contribute to inflammasome activation and pyroptosis. For instance, *srb9*Δ/Δ mutant hyphae, which show reduced surface-exposed β-1,3-glucan, also exhibit reduced ability to cause IL-1β secretion and macrophage death post-phagocytosis [14]. Furthermore, fungal cell wall remodelling within the macrophage phagosome and exposure of glycosylated mannoproteins were required to trigger macrophage pyroptosis [7,12]. Additionally, increased biosynthesis of fungal plasma membrane ergosterol during the yeast-to-hypha transition within phagosomes and its association with the outer mannoprotein layer have been shown to trigger inflammasome activation and pyroptosis [15]. Fungal cell wall remodelling was also observed to be dispensable for the priming step but crucial for inflammasome activation and macrophage pyroptosis, in the absence of phagolysosomal rupture [7]. However, how the signal moves from the phagosome to the cytoplasm to activate the cytosolic NLRP3 remains unclear. Indeed, whether the fungal moieties interact with membrane-bound receptors and/or enter the cytosol to trigger inflammasome activation has not yet been determined.

Apart from cell wall components, secreted molecules from *C*. *albicans* can also act as triggers of inflammasome activation. For instance, clathrin-dependent internalisation of secreted aspartyl proteinases (Sap2p and Sap6p) by human mononuclear phagocytes induces an early cascade of events (potassium efflux, ROS production, and lysosomal damage) leading to canonical NLRP3/caspase-1 activation and IL-1β/IL-18 production [16]. Additionally, studies with murine macrophages reveal that Sap-induced type I interferon production activated a noncanonical/caspase-11 inflammasome, which enhanced caspase-1 activation and cytokine production [16]. Furthermore, secretion of the peptide toxin candidalysin can also induce NLRP3/ASC/caspase-1 assembly and IL-1β maturation in human and murine macrophages via a mechanism requiring potassium efflux [17]. Candidalysin is secreted by hyphae, and phagocytes can be exposed to hyphae either pre- or post-phagocytosis. However, experiments conducted in the presence of cytochalasin D have shown inhibition of candidalysin-induced inflammasome activation, suggesting that toxin internalisation is required [17]. Thus, although a rapidly growing body of literature has begun to unravel the regulation and molecular mechanisms responsible for inflammasome activation during *Candida* infection, much remains to be learned, and further investigations are required for a better understanding of the precise mechanistic details.

## Inflammasomes are immunological weapons in the fight against *C*. *albicans* infection

In vivo studies have confirmed that inflammasomes are critical for mounting an effective anti-*Candida* response and in restricting fungal growth and dissemination. Using a murine model of oropharyngeal candidiasis (OPC) and *Il1r*^-/-^, *Nlrp3*^-/-^, *Asc*^-/-^, and *caspase-1*^-/-^ mice, Hise and colleagues showed that inflammasome components were critical for controlling *C*. *albicans* burdens in tongue tissue, preventing dissemination to the kidneys and enhancing host survival [6]. In a follow-up study, NLRC4 inflammasome activation was also demonstrated to prevent OPC and early systemic dissemination of *C*. *albicans* infection by coordinating both innate and adaptive immune responses [18]. Neutrophils are essential for innate immunity and resistance to fungal pathogens, and IL-1β participates in driving neutrophil recruitment at the site of infection. Neutrophil influx into the *Nlrc4*^-/-^ tongue was drastically reduced compared to either wild-type (wt) or *Nlrp3*^-/-^ mice, and NLRC4 activation was crucial for the trafficking of neutrophils to the site of active *Candida* infection. Furthermore, antimicrobial peptides are important effector molecules of innate immunity that disrupt pathogen function and act as regulators of inflammation. Interestingly, the expression of antimicrobial peptides in the buccal mucosal tissue of *Nlrc4*^-/-^, *Nlrp3*^-/-^, and *Asc*^-/-^ mice was strongly reduced compared to wt mice following *C*. *albicans* infection. Similarly, mucosal IL-17 responses to *Candida* were dependent on both NLRP3 and NLRC4 [18]. OPC was more severe in *Nlrc4*^-/-^ mice compared with either wt or *Nlrp3*^-/-^ mice, suggesting that NLRC4 plays a more prominent role than NLRP3 against oral infection. In addition, murine bone marrow chimaera experiments showed that OPC is controlled by NLRC4 functioning in the stromal and epithelial compartment and by NLRP3 functioning in hematopoietic-derived inflammatory cells, whilst protection against disseminated fungal infection was driven by NLRP3 functioning in both hematopoietic and stromal cell lineages [18].

Currently, the fungal components that activate inflammasomes at mucosal surfaces are unclear, but oral epithelial cells are known to secrete IL-1β in response to candidalysin [19]. Notably, IL-1β release may occur through epidermal growth factor receptor (EGFR) activation [20] and drives the proliferation of innate IL-17+TCRαβ+lymphocytes in tongue tissue [21], which are essential for the resolution of murine OPC [22]. These data strongly suggest a central role for candidalysin in inflammasome-mediated defences against OPC. Epithelial inflammasomes may also be activated through the EphA2 receptor, since oral tissues of *EphA2*^−/−^ mice expressed lower levels of IL-1β, interferon gamma (IFN-γ), and IL-17A and harboured higher fungal burdens and greater fungal dissemination to the liver [23].

Inflammasome activation is also critical for protection against (intravenous) disseminated *C*. *albicans* infection. *Nlrp3*^-/-^ mice rapidly succumb to *C*. *albicans* infection compared with wt mice [8] and are associated with significantly increased fungal burdens in the kidneys, liver, spleen, and lungs [8,10]. Likewise, *Asc*^-/-^ and *Caspase-1*^-/-^ mice were more susceptible to disseminated candidiasis, with bone marrow–derived dendritic cells releasing significantly reduced levels of IL-1β and IL-18 following *C*. *albicans* challenge [24]. In addition, impaired production of IL-17 and IFN-γ was observed in *C*. *albicans*–challenged splenocytes isolated from *Il-1β*^-/-^ and *Il-18*^-/-^ mice, respectively [24].

**Fig 1:**
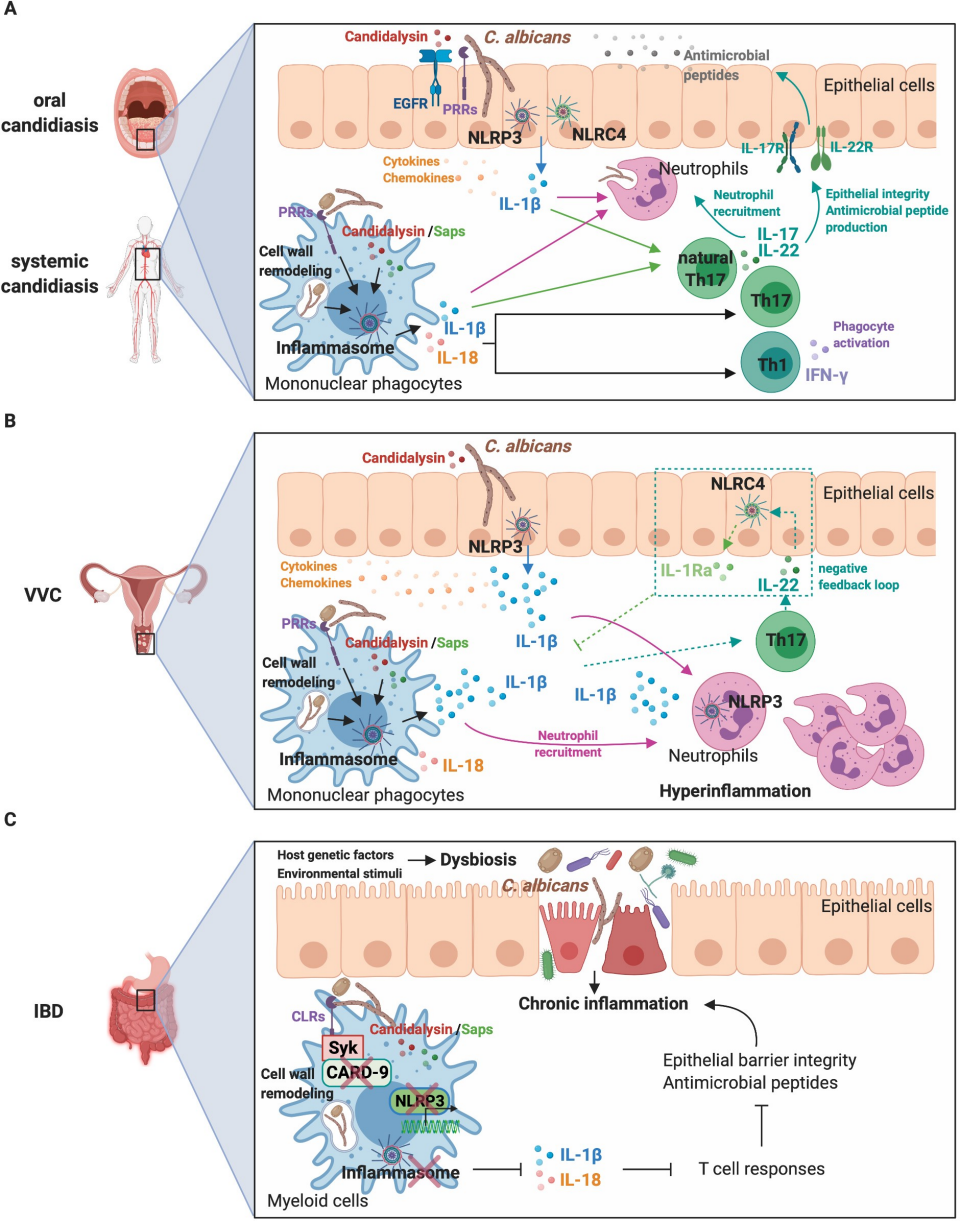
Interplay between *C*. *albicans*, inflammasomes, and the host immune system during infection. **(A)** NLRP3 and NLRC4 inflammasome activation in epithelial and myeloid cells and the release of IL-1β and IL-18 during oral and systemic candidiasis are instrumental components of the inflammatory response, ultimately orchestrating both innate and adaptive immunity to eliminate the fungus. **(B)** During VVC, hyphae- and hypha-associated virulence factors induce a powerful NLRP3 inflammasome and inflammatory response that drives the recruitment of large numbers of neutrophils to the site of inflammation. Host genetic variations in inflammasome and other pathways likely influence VVC susceptibility and hyperreactivity to the fungus. An imbalance of NLRP3 inflammasome activation and its IL-22/NLRC4/IL-1Ra negative feedback pathway also contributes to hyperinflammation and disease pathogenesis. **(C)** A combination of environmental (e.g., diet, antibiotics, and antifungals) and host genetic factors can promote dysbiosis and loss of intestinal immune homeostasis. Impaired mucosal barrier function and dysregulated antimicrobial immune responses result in uncontrolled chronic inflammation. In the genetically susceptible host, impaired inflammasome-mediated anti-*Candida* responses contribute to intestinal inflammation and IBD pathogenesis. CARD-9, caspase recruitment domain-containing protein 9; CLRs, C-type lectin receptors; EGFR, epidermal growth factor receptor; IBD, inflammatory bowel disease; IFN-γ, interferon gamma; IL, interleukin; IL-1Ra, IL-1 receptor antagonist; NLRC4, NOD-like receptor family CARD domain-containing protein 4; NLRP3, NOD-like receptor family pyrin domain-containing 3; PRRs, pattern recognition receptors; Saps, secreted aspartyl proteinases; Syk, spleen tyrosine kinase; Th, T-helper; VVC, vulvovaginal candidiasis.

Together, these findings suggest that the activation of inflammasomes plays a prominent role in driving protective innate and adaptive antifungal immune responses during mucosal and invasive *Candida* infections (Fig 1A). However, the precise role of *C*. *albicans*–triggered pyroptosis has yet to be elucidated. Importantly, a recent study has shown that pyroptosis occurs in vivo in the kidneys of infected mice during the early stages of infection [15]. Furthermore, neutrophil recruitment in the kidneys of infected mice was dependent on *C*. *albicans* mutant strains capable of inducing inflammasome activation and pyroptosis [7]. Thus, although pyroptosis may represent an immune evasion strategy to overcome killing by macrophages, this inflammatory form of programmed cell death may also favour neutrophil recruitment to eliminate the pathogen.

## NLRP3 inflammasome: A double-edged sword in Candida infection

Whilst inflammasome activation is generally considered beneficial for mounting protective anti-*Candida* responses, it may also be detrimental and drive immunopathology, as seems to be the case in vulvovaginal candidiasis (VVC). Symptomatic VVC infection is linked to immune hyperreactivity to the fungus and characterised by significant vaginal neutrophil infiltration, NLRP3 inflammasome activation, and increased cytokine production. Notably, polymorphisms and variable number tandem repeats in the *NLRP3* gene are associated with VVC [25,26]. Likewise, overexpression of NLRP3, caspase-1, and elevated IL-1β secretion are observed in vaginal epithelial cells isolated from symptomatic VVC patients [27]. In murine studies, neutrophil numbers and IL-1β levels were reduced in vaginal lavage fluid of *Nlrp3*^-/-^ mice challenged with *C*. *albicans* [28]. Interestingly, both filamentation [29] and candidalysin secretion [30] are critical in driving inflammation (including IL-1β), neutrophil recruitment, and pathology in VVC, suggesting a primary role for candidalysin in vulvovaginal immunopathogenesis (Fig 1B).

IL-22 also appears to play a role in VVC. A functional single nucleotide polymorphism (SNP) in the human *IL-22* gene was found to be associated with a decreased risk for VVC and correlated with increased IL-22 expression [31]. Borghi and colleagues demonstrated that an IL-22/NLRC4/IL-1 receptor antagonist (IL-1Ra) axis controls NLRP3 activation during *Candida* infection [32]. In this model, IL-22 production via the aryl hydrocarbon receptor (AhR) activates epithelial NLRC4, which, in turn, restrains NLRP3 activity by inducing sustained production of IL-1Ra. Similarly, NLRC4, but not NLRP3 expression, was increased by IL-22 in response to *Candida* in human vulvovaginal A431 cells. Moreover, high levels of IL-1β and low levels of IL-1Ra and IL-22 were observed in vaginal fluids of patients with recurrent VVC, suggesting that defective production of IL-1Ra and IL-22 and the impairment of this axis may contribute to disease pathogenesis [32] (Fig 1B). These data highlight the importance of regulating inflammasome activation to fine tune inflammatory processes during *C*. *albicans* infection. However, whether the pro-inflammatory nature of *C*. *albicans*–induced pyroptosis [7] contributes to disease pathogenesis is still largely unknown and is thus an important direction for future research.

## *Candida*, inflammasomes, and inflammatory bowel disease

Inflammatory bowel disease (IBD) is characterised by chronic inflammation of the gastrointestinal (GI) tract, which includes ulcerative colitis (UC) and Crohn’s disease (CD). Notably, alterations in the biodiversity and composition of fungal microbiota, with an expansion of *C*. *albicans*, are observed in IBD patients [33,34]. Although the cause and the exact mechanism of IBD pathogenesis remain to be elucidated, environmental stimuli (e.g., diet, antibiotics, and antifungals), imbalanced interactions with commensal gut microbes, including fungi, and aberrant immune responses appear to contribute to the development of the chronic intestinal inflammation in genetically susceptible individuals [35]. Interestingly, IBD is associated with SNPs in dectin-1, which is a critical CLR required for an effective antifungal immune response [36]. Consistently, dextran sulfate sodium (DSS)-induced colitis was more severe following *Candida tropicalis* supplementation in *dectin-1*^*-/-*^ mice. In addition, fluconazole treatment reduced colitis symptoms, inflammatory cell infiltration, and production of IL-17 and IFN-γ by T cells, confirming that impaired antifungal immunity leads to increased disease severity [36]. Similarly, higher levels of neutrophil infiltration, colon lesions, and IL-1β expression were observed in DSS-treated mice following GI colonisation with *C*. *albicans* [37].

IBD is also associated with SNPs in *CARD-9* [38], and mice lacking CARD-9 or the kinase Syk show reduced inflammasome activation and IL-18 secretion during azoxymethane (AOM)–DSS-induced colitis-associated colon cancer [39]. These mice also exhibited reduced IFN-γ production by T cells and increased inflammation and epithelial hyperplasia. Notably, similar events occurred when wt mice were depleted of commensal fungi and disease severity significantly ameliorated by exogenous IL-18 supplementation. Furthermore, CARD-9 and Syk were required for caspase-1 activation and IL-18 release in bone marrow–derived myeloid cells in response to *C*. *albicans*. Thus, inflammasome-mediated IL-18 release through the activation of Syk/CARD-9 signalling by commensal gut fungi preserves epithelial barrier function, promotes CD8+ T cell responses, and ultimately, restrains colitis and colon tumorigenesis [39]. Interestingly, CD is also associated with SNPs in *NLRP3* and concomitant reduction in IL-1β production [40]. Indeed, whilst the exact role of NLRP3 in IBD is not yet fully elucidated, increasing evidence has suggested that appropriate inflammasome activation has a protective effect in colitis and colitis-associated carcinogenesis [41]. For instance, *Nlrp3*^-/-^, *Asc*^-/-^, and *caspase-1*^-/-^ mice were observed to be highly susceptible to DSS-induced colitis. Furthermore, these mice also exhibited commensal overgrowth and bacteremia, increased DSS-induced morbidity and lethality, and an exaggerated immune response, which may further worsen disease severity [42]. Similarly, a pathophysiological model linking impaired anti-*Candida* responses, including an impaired NLRP3 inflammasome activity, and the development of CD has been suggested. This model supports a scenario whereby the progression of IBD in genetically susceptible individuals results from a vicious cycle: inflammation and abnormalities in immune regulation promote proliferation and mucosal invasion by *C*. *albicans*, which, in turn, exacerbates the inflammatory process and tissue damage as a consequence of immune dysregulation [43].

Although animal models cannot fully reflect human diseases, they have greatly contributed to our understanding of the mechanism of IBD and the potential contribution of gut fungi and dysregulated immune responses to IBD pathogenesis. Interestingly, the anti-IL-17A monoclonal antibody secukinumab was ineffective in the treatment of CD in a randomised, double-blind, placebo-controlled proof-of-concept study and highlighted adverse effects in patients including a higher frequency of *Candida* infections compared to placebo [44]. This result further suggests that impaired anti-*Candida* immunity, including defective inflammasome-driven responses, may play a role in human IBD. Nonetheless, further investigation is required to confirm this model and the relationship between *Candida*, inflammasome dysregulation, and GI inflammation (Fig 1C).

## Inflammasomes as therapeutic targets in *Candida* disease

Inflammasome activation plays a pivotal role in antifungal immune responses and is a tightly regulated process. Dysregulation of the inflammasome can lead to host damage and excessive inflammation, with inflammasome hyperactivation being a central player in several human autoinflammatory and autoimmune diseases. This has encouraged efforts to identify potent and specific ways to interfere with inflammasome activation or the activity of inflammasome-dependent cytokines [1]. Hence, targeting inflammasome pathways may provide novel and effective therapeutic approaches to neutralise potent inflammatory mediators that exacerbate the pathogenesis of *C*. *albicans* infection, as in the case of VVC. Notably, administration of recombinant IL-1Ra (anakinra) can reduce NLRP3-driven inflammation and protect against infection in mouse models of *Candida* vaginitis [32]. Furthermore, the NLRP3 inhibitor MCC950 and the adenosine triphosphate (ATP)-sensitive potassium channel inhibitor glyburide, which also inhibits inflammasome activation, significantly reduced IL-1β release by the human monocytic cell line THP-1 following *Candida* exposure or stimulation with candidalysin [45]. Likewise, intravaginal inoculation of glyburide in mice prior to *C*. *albicans* infection reduced neutrophil infiltration and IL-1β production in vaginal lavage fluid [28]. These findings demonstrate the potential role for inflammasome inhibitors or anti-IL-1β treatment in the control of hyperinflammation-driven *C*. *albicans* diseases. Future work will determine whether such a therapeutic approach can be translated to clinical care.
